# Synthetic acid stress-tolerance modules improve growth robustness and lysine productivity of industrial *Escherichia coli* in fermentation at low pH

**DOI:** 10.1186/s12934-022-01795-4

**Published:** 2022-04-22

**Authors:** Xurong Yao, Peng Liu, Bo Chen, Xiaoyan Wang, Fei Tao, Zhanglin Lin, Xiaofeng Yang

**Affiliations:** 1 School of Biology and Biological Engineering, South China University of Technology, 382 East Outer Loop Road, University Park, Guangzhou, 510006 Guangdong China; 2 COFCO Nutrition & Health Research Institute, Beijing, 102209 China; 3 State Key Laboratory of Microbial Metabolism, Joint International Research Laboratory of Metabolic and Developmental Sciences, School of Life Sciences and Biotechnology, Shanghai Jiao Tong University, Shanghai, 200240 China

**Keywords:** Synthetic modules, Acid tolerance, Acid-responsive promoter library, Stepwise screening, Lysine production, *Escherichia coli*

## Abstract

**Background:**

During fermentation, industrial microorganisms encounter multiple stresses that inhibit cell growth and decrease fermentation yields, in particular acid stress, which is due to the accumulation of acidic metabolites in the fermentation medium. Although the addition of a base to the medium can counteract the effect of acid accumulation, the engineering of acid-tolerant strains is considered a more intelligent and cost-effective solution. While synthetic biology theoretically provides a novel approach for devising such tolerance modules, in practice it is difficult to assemble stress-tolerance modules from hundreds of stress-related genes.

**Results:**

In this study, we designed a set of synthetic acid-tolerance modules for fine-tuning the expression of multi-component gene blocks comprising a member of the proton-consuming acid resistance system (*gadE*), a periplasmic chaperone (*hdeB*), and reactive oxygen species (ROS) scavengers (*sodB* and *katE*). Directed evolution was used to construct an acid-responsive *asr* promoter library, from which four variants were selected and used in the synthetic modules. The module variants were screened in a stepwise manner under mild acidic conditions (pH 5–6), first by cell growth using the laboratory *Escherichia coli* strain MG1655 cultured in microplates, and then by lysine production performance using the industrial lysine-producing *E. coli* strain MG1655 SCEcL3 cultured first in multiple 10-mL micro-bioreactors, and then in 1.3-L parallel bioreactors. The procedure resulted in the identification of a best strain with lysine titer and yield at pH 6.0 comparable to the parent strain at pH 6.8.

**Conclusion:**

Our results demonstrate a promising synthetic-biology strategy to enhance the growth robustness and productivity of *E. coli* upon the mildly acidic conditions, in both a general lab strain MG1655 and an industrial lysine-producing strain SCEcL3, by using the stress-responsive synthetic acid-tolerance modules comprising a limited number of genes. This study provides a reliable and efficient method for achieving synthetic modules of interest, particularly in improving the robustness and productivity of industrial strains.

**Supplementary Information:**

The online version contains supplementary material available at 10.1186/s12934-022-01795-4.

## Background

Microbial production of chemicals and fuels is a growing industry, and has been playing an increasingly significant role in the global economy, e.g., it accounts for more than 2% of the national gross domestic product (GDP) in the USA [1]. Microorganism can encounter multiple stressing conditions during industrial bioprocesses, such as high and low temperatures, acid, oxidative and osmotic stresses, and nutritional starvation, that negatively affect cell growth and productivity [2–4]. For example in amino acids or organic acids production, although the issue can be addressed by adding base at the fermentation broth, however, it would increase the downstream separation costs, the use of neutralizing reagents, and the waste streams [5–7]. In the large scale fermentation, the addition of base can also cause pH fluctuation above the neutral pH, and bring pH stresses to the cells in the opposite direction [4]. Thus, engineering of cellular tolerance toward environmental stress has been a long-standing critical issue for bioprocess economics.

Over the years, several genome-wide evolution engineering strategies have been widely used to enhance the stress resistance for microbial cells [8]. Among them, adaptive laboratory evolution (ALE) is a traditional and commonly used strategy to enhance stress tolerance [9–11]. However, it is difficult to identify the mutations associated with the desired phenotype [12, 13]. A second genome-wide engineering strategy, namely global transcription machinery engineering (gTME), has also been developed for engineering global transcription regulators, like CRP, RpoD, IrrE and H-NS [12, 14–16]. A number of strains with improved stress resistance has been reported for this strategy, but their industrial application has been limited, likely because of their inefficient consumption of cellular energy and resources resulting from the simultaneous perturbation of hundreds of non-essential genes [17, 18]. More recently, clustered regularly interspaced short palindromic repeats (CRISPR) enabled trackable genome engineering (CREATE) and synthetic chromosome rearrangement and modification by loxP-mediated evolution (SCRaMbLE) have been developed for bacteria or yeast [19, 20]. These new tools are being tested [8].

Synthetic biology offers a useful approach to design synthetic acid-tolerance modules or other stress-tolerance modules based on stress resistance mechanisms and stress-tolerance genes mining, in a systematic, predictable, and transferrable fashion [21, 22]. To date, a handful of studies showed the feasibility of employing different combinations of multiple stress-tolerance genes to assemble synthetic stress-tolerance modules that enhance cell tolerance towards different stresses. For example, in a study on yeast SyBE005, stress sensing promoters were used to regulate the superoxide dismutase gene *SOD1*, the glutamate-cysteine ligase gene *GSH1*, the glutathione-disulfide reductase gene *GLR1* of the antioxidant system, the glucose-6-phosphate dehydrogenase gene *ZWF1* of the glycolysis pathway, and the acetyl-CoA synthase gene *ACS1*, which is involved in the acetyl-CoA synthesis, leading to an improved ethanol titer by 49.5% at the shake flask scale [23]. In another study on *Escherichia coli* DH10B, the DNA binding protein gene *hu*, which is involved in DNA protection, the RNA binding protein gene *rbp*, which is involved in RNA protection, and the ATP-dependent serine protease gene *clpP*, which is involved in misfolded protein degradation, were overexpressed, leading to an increase in survival rate of over 600-fold for the best strain upon acid shock at pH 1.9 [24]. It is noteworthy that many studies on acid tolerance have focused on cell survival under extreme pH conditions, however efficient cell growth and productivity under moderate acidic conditions are more valuable for industrial applications [15, 25].

The acid resistance (AR) of microorganisms is a complex polygenic trait, e.g. *E. coli* uses a range of physiological, metabolic, and proton-consuming AR mechanisms to survive acid stress [26]. In our previous studies, which were based on two different schemes, i.e. overexpressing a mutated global transcriptional factor H-NS [15], or the small RNA (sRNA) DsrA together with the sRNA chaperone Hfq [25], we found that, in terms of growth at moderate acidic pH (pH 4.5), a same set of three mechanisms, including the proton-consuming acid resistance system, periplasmic chaperones, and reactive oxygen species (ROS) scavengers, were the most important contributors for the acid-tolerant phenotype. We therefore suspected that genes involved in these three systems may be the most important factors that control acid resistance in *E. coli*.

In this study, we designed a set of synthetic acid-tolerance modules to improve the acid resistance of *E. coli* under mild acidic condition, by fine-tuning the expression of the genes involved in the proton-consuming acid resistance system (*gadE*), a periplasmic chaperone (*hdeB*) and ROS scavengers (*sodB* and *katE*) which participate in the aforementioned three stress resistance systems. The transcriptional regulator GadE is the key activator of the proton consumption system AR2 [27–31]. Periplasmic chaperones HdeA and HdeB are both known to prevent periplasmic proteins aggregation at low pH, the latter being more efficient under mild acid stress (pH 4–6) [32, 33]. Superoxide dismutase and catalase catalyze the conversion of superoxide radical to hydrogen peroxide, which is then further converted into water and oxygen [34, 35]. A previous study found that overexpressing *sodB* and *katE* enhanced the titer of 5-aminolevulinic acid by 117% in a 5-L fermenter [36].

With the goal of achieving a just-enough and just-in-time gene expression, we used directed evolution to construct an acid-responsive promoter pool with a gradient of strengths, from which four promoter variants with different strengths were assembled with the four genes *gadE*, *hdeB*, *sodB* and *katE* into synthetic acid-tolerance modules. The different modules variants were cloned into the laboratory *E. coli* strain MG1655, and were then screened and analyzed for cell growth at pH 5.0. Subsequently, 33 synthetic acid-tolerance modules were evaluated in an industrial lysine-producing *E. coli* strain*,* MG1655 SCEcL3, using multiple 10-mL micro-bioreactors and 1.3-L parallel bioreactors, resulting in a best strain with improved l-lysine yield at pH 6.0 compared with the parent strain at pH 6.8. This work provides an example of semi-rational design of industrial *E. coli* strains for improving the production robustness at low fermentation pH.

## Results

### Construction and characterization of the acid-responsive promoter library

**Fig. 1:**
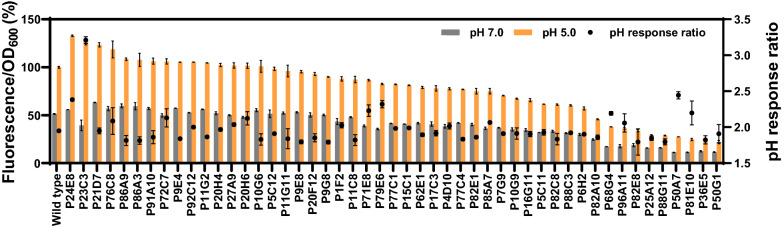
Relative strengths of the synthetic *asr* promoter library entries, compared to the wild type *asr* promoter. The black dots represent the pH response ratio of the fluorescence signals obtained in LBG medium at two different pH (pH 5.0 vs pH 7.0). Each experiment was performed in three biological replicates

As the promoter, we chose the acid-responsive acid shock RNA (*asr*) promoter [37, 38], and randomized the 9 bp spacer region between the PhoB box and the − 10 nucleotide of the wild type *asr* sequence using degenerated primers (Additional file 1: Fig. S1). This region has been reported to affect the promoter strength [39, 40]. The variants were cloned into the reporter plasmid pACYC184 [41], upstream of a *mCherry* gene coding for a red fluorescence protein that is stable between pH 4–8 [42], and screened in *E. coli* to determine the pH response ratio in the fluorescence signals in LBG medium at two pH values (pH 5.0 vs pH 7.0). About 6000 clones were randomly picked for a first round of screening. It is noteworthy that many of these variants lost the activity or acid-responsiveness [40, 43]. 219 positive clones were selected for re-screening in triplicate and sequencing, from which 177 unique variants were found (Additional file 1: Fig. S2). Finally, 49 *asr* promoter variants with strengths ranging from 22 to 134% of the wild type (at pH 5.0), and the ratio above 1.8 were obtained (Fig. 1 and Additional file 2: Table S1). For the wild type, this ratio was 1.95. We also evaluated the mutations occurred in these variants (Additional file 1: Fig. S3). Consistent with the wild type *asr* promoter, adenine was the dominant nucleotide at positions − 17 and − 13 with frequencies of 61% and 57%, respectively. Differently from the wild type promoter, adenine was also the dominant nucleotide at positions − 16, − 15 and − 14, with frequencies of 53%, 55%, and 57%, respectively.

Stepwise screening is an efficient strategy for engineering stress tolerant strain [44, 45]. Our study adopted the following protocol: (1) first a cell growth-guided high-throughput screening at mild acidic pH (5.0) performed using a laboratory *E. coli* strain as the model organism, a basic fermentation medium and the automated turbidimeter Bioscreen C, which can handle 200 strains at once; (2) then a medium-throughput screening guided by lysine productivity performed using an industrial strain, an industrial medium and the small-volume bioreactor Micro-Matrix which can handle 24 strains at once; (3) finally the industrial strain variants were analyzed in a laboratory-scale parallel-bioreactor system, which can handle 8 strains at once. We surmise that although cell growth is not necessarily correlated with high production, it is a prerequisite for high production [46, 47]. Thus, the cell growth assay helped to select candidates for high production. Indeed, the most productive strains we selected did also perform well in cell growth (see below).

### Application of the acid-responsive promoter library to the construction of acid-tolerance modules

**Fig. 2:**
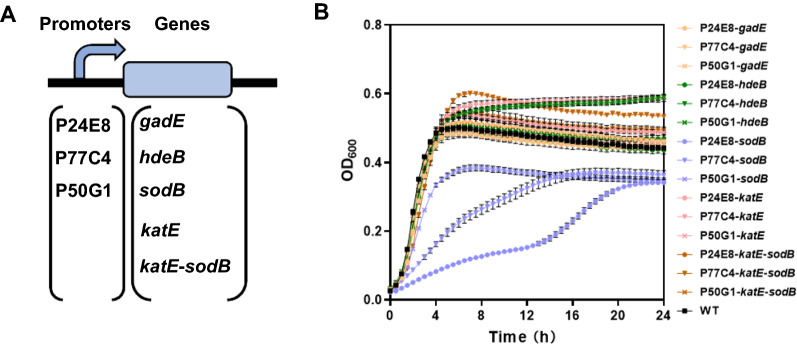
Schematic diagram and cell growth of the modules for the strains overexpressing *gadE*, *hdeB*, *sodB*, *katE* or *katE*-*sodB*. Schematic diagram (**A**). Cell growth of *E. coli* strains overexpressing *gadE*, *hdeB*, *sodB*, *katE,* or *katE-sodB* under the control of different *asr* variants and wild type *E. coli* MG1655 (WT) cultured for 24 h in LBG at pH 5.0 (**B**). The OD_600_ was measured every 30 min. Each experiment was performed in three biological replicates

In order to investigate the effect of selected genes on the acid tolerance of *E. coli* under mild acidic condition (pH 5.0), we individually overexpressed the four genes *gadE*, *hdeB*, *sodB*, *katE,* and the *katE-sodB* cascade, under the control of three different *asr* promoter variants, P24E8, P77C4 and P50G1, using plasmid pACYC184 backbone (Fig. 2A). The relative strengths of these three promoter variants at pH 5.0 were 133%, 77% and 22% of the wild type *asr* promoter, and the corresponding pH response ratios were 2.38, 1.83, and 1.91, respectively.

**Fig. 3:**
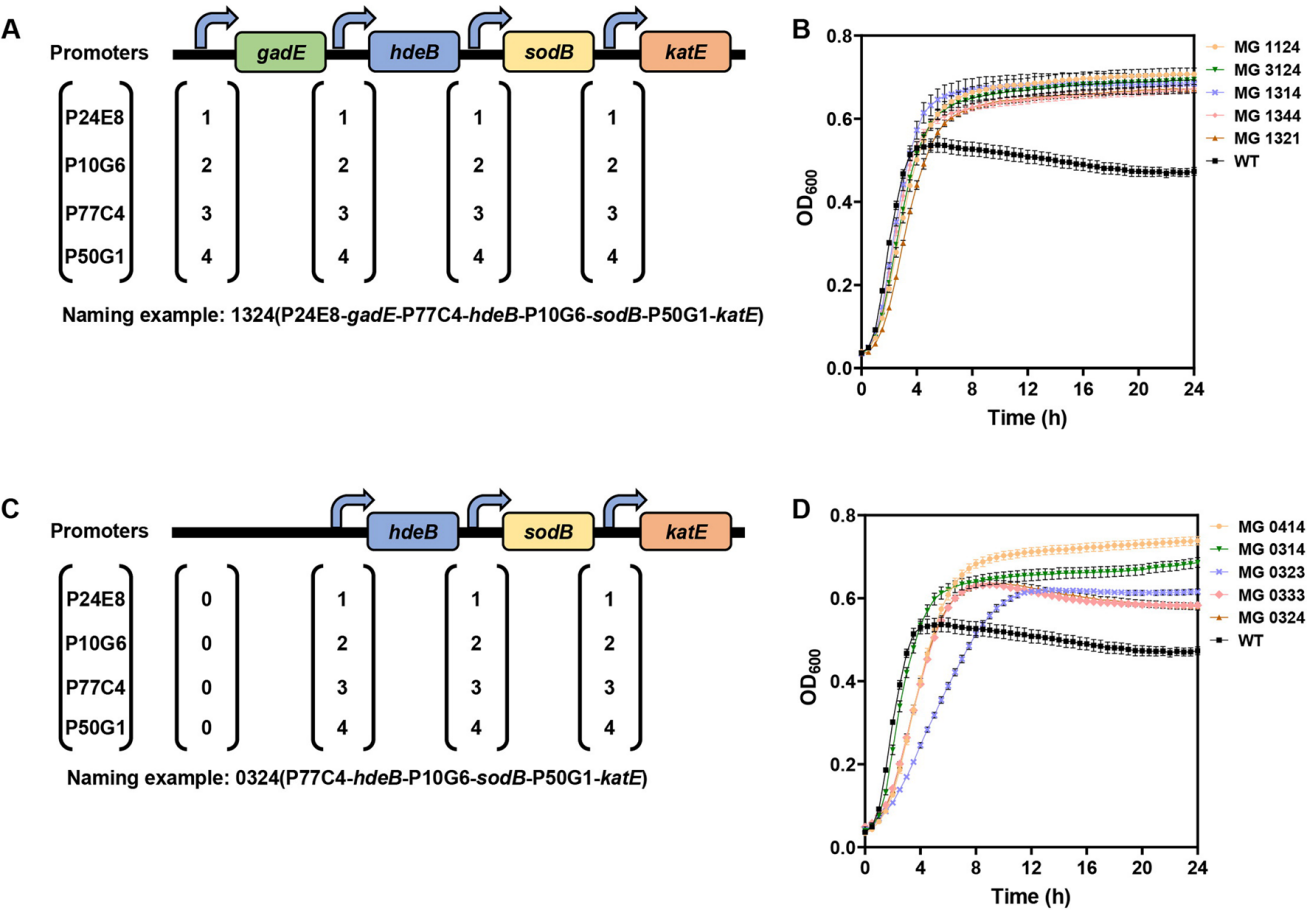
Schematic diagram of the modules and the naming rules for the libraries entries produced in this study and cell growth of the top five strains harboring modules of the libraries. Schematic diagram of the modules in the *gadE*-*hdeB*-*sodB*-*katE* library (**A**) and in the *hdeB*-*sodB*-*katE* library (**C**). Naming rules. Each name consists of a code in which the digit position i.e., first, second, third and fourth, indicates the gene to which a promoter is associated i.e., *gadE*, *hdeB*, *sodB*, *katE*, respectively. The promoter identity is indicated with a different number: (1) P24E8; (2) P10G6; (3) P77C4; (4) P50G1; 0 indicates the lack of the gene in the module. Cell growth of the top five recombinant strains in the *gadE*-*hdeB*-*sodB*-*katE* library (**B**), in the *hdeB*-*sodB*-*katE* library (**D**) and wild type *E. coli* MG1655 (WT) cultured for 24 h in LBG at pH 5.0. The OD_600_ was measured every 30 min. Each experiment was performed in three biological replicates

As shown in Fig. 2, the final OD_600_ of P24E8-*gadE*, P77C4-*gadE* and P50G1-*gadE* were 100%, 104% and 102% of that of the wild type strain (*E. coli* MG1655), respectively, indicating that *gadE* alone conferred little acid tolerance to *E. coli*. The overexpression of *hdeB* enhanced the acid resistance, as the final OD_600_ of P24E8-*hdeB*, P77C4-*hdeB* and P50G1-*hdeB* at pH 5.0 reached 99%, 106% and 134% of that of the wild type strain, respectively. The overexpression of *katE and katE*-*sodB* improved the cell growth at pH 5.0, as the final OD_600_ of P24E8-*katE*, P77C4-*katE* and P50G1-*katE* reached 105%, 133% and 110% of that of the wild type strain, respectively, and the final OD_600_ of P24E8-*katE*-*sodB*, P77C4-*katE*-*sodB* and P50G1-*katE*-*sodB* were 106%, 121% and 112% of that of the wild type strain, respectively. This indicated that *hdeB* and *katE*-*sodB* can enhance acid resistance of *E. coli* in mild acidic conditions under the control of the selected *asr* promoter variants. On the other hand, the overexpression of *sodB* alone did not enhance the final OD_600_ of the strains with any of the promoters, and the lag phase was prolonged. These results are consistent with the fact that the enzyme SodB produces H_2_O_2_ which might actually damage the intracellular environment [34, 35]. Although the final OD_600_ of the strain harboring P77C4-*katE*-*sodB* was lower than that of the strain harboring P77C4-*katE*, given that *sodB and katE* forms a cascade relationship [36], and that the function of this cascade might not be fully realized during this initial screening step using the laboratory strain *E. coli* MG1655, we thus included this cascade in the following analyses, but used separate promoters in order to individually fine tune the two genes (Fig. 3).

### Construction, selection and analysis of acid-tolerant recombination strains

We combined the four genes with the four *asr* promoter variants P24E8, P10G6, P77C4 and P50G1. P10G6 was included for the fine-tuning of the four genes into a library of synthetic acid-tolerance modules (*gadE-hdeB-sodB-katE*) (Fig. 3A). The relative strength of P10G6 was 100% of the wild type *asr* promoter, with a pH response ratio of 1.83.

The four-gene synthetic acid-tolerance module library had 256 possible distinct variants. We randomly picked 832 clones in order to achieve approximately a 95% coverage of the library, and we characterized them by cell growth assay using an automated turbidimeter (Bioscreen C) [48]. Among the clones selected, 244 unique strains were identified, corresponding to a 95.3% coverage of the library. The compositions of the *asr* promoters in these 244 strains are shown in Additional file 3, along with the respective final OD_600_ ratios and the maximum specific growth rate ratios, compared with the wild type strain. The standard deviations of the final OD_600_ ratio and maximum specific growth rate ratio of each strain were mostly below 5%, with an average of 2.3% for the final OD_600_ ratio and 1.5% for the maximum specific growth rate ratio. In terms of growth, 24 strains showed a final OD_600_ higher than 120% of that of the wild type strain. The maximum specific growth rates were 55–103% of that for the wild type strain, indicating that the overexpression of the four genes also brought burden to *E. coli*. The growth curves of the top five recombinant strains and the wild type at pH 5.0 are shown in Fig. 3B. The final OD_600_ of the five best strains MG 1124, MG 3124, MG 1314, MG 1344 and MG 1321 were 0.71, 0.69, 0.68, 0.67 and 0.67, which were 151%, 147%, 145%, 143% and 143% of the OD_600_ of the wild type strain (i.e., 0.47), respectively (see Fig. 3 for the naming rules applied to the strains used in this study). Comparison of these data with the OD_600_ obtained when the genes were separately overexpressed showed that the four-gene modules could further enhance the acid tolerance of *E. coli* compared with the individual overexpressed *gadE*, *hdeB*, *sodB, katE*, or *katE*-*sodB*. The maximum growth rates of the five best strains mentioned above were 0.99 h^−1^, 1.05 h^−1^, 1.12 h^−1^, 1.10 h^−1^, and 0.91 h^−1^, which were 73%, 78%, 83%, 82% and 67% of the growth rate of the wild type strain (i.e., 1.35 h^−1^), respectively.

### Analysis and modification of the synthetic acid-tolerance modules

To understand the contribution of each gene in the four-gene modules for the acid tolerant phenotype, a multi-factorial analysis of variance (ANOVA) was performed [49] (Additional file 2: Table S2). In this analysis, the four genes were set as the factors, the four *asr* promoter variants were set as the ordinal levels, and the final OD_600_ ratios were set as the responses. The result showed that *hdeB* and *sodB* were significantly correlated to the final OD_600_ (p < 0.05), but the p-value for *katE* (0.056) was slightly above the statistical threshold. Surprisingly, the contribution of *gadE* to growth was not significant, with a p-value of 0.862.

We then constructed a new recombination strain library co-expressing just the three genes that showed contribution to the phenotype in the ANOVA test (*hdeB*, *sodB* and *katE*), but with the same four *asr* promoter variants used for the four-gene modules (Fig. 3C). Among the 76 clones that were randomly picked and characterized by cell growth assay, 31 unique strains were identified (Additional file 4). The standard deviations of the final OD_600_ ratio and maximum specific growth rate ratio of each strain were mostly below 5%, with an average of 1.9% for the final OD_600_ and 1.5% for the maximum specific growth rate ratio. The growth curves of the top five recombinant strains at pH 5.0 are shown in Fig. 3D, and the final OD_600_ of these five strains, namely MG 0414, MG 0314, MG 0323, MG 0333 and MG 0324, were 0.74, 0.69, 0.62, 0.58, and 0.58 respectively, which were 157%, 147%, 132%, 123%, and 123% of the OD_600_ of the wild type strain (i.e., 0.47). Among these strains, MG 0414 and MG 0314 grew better than MG 1124 (i.e., the strain with the highest OD_600_ among the strains in the *gadE*-*hdeB*-*sodB*-*katE* module library). The maximum specific growth rates of MG 0414, MG 0314, MG 0323, MG 0333 and MG 0324 were 0.77 h^−1^, 1.11 h^−1^, 0.59 h^−1^, 0.69 h^−1^, and 0.72 h^−1^, which were 78%, 112%, 60%, 70%, and 73% of that of MG 1124 (i.e., 0.99 h^−1^). The final OD_600_ of MG 0414 and MG 0314 were higher than their corresponding four-gene counterparts harboring also *gadE*. These results indicated that for the lab strain MG1655 used in this step, the *hdeB*-*sodB*-*katE* modules were sufficient to confer acid tolerance to the cells at pH 5.0, in terms of final OD_600_, or the further overexpression of *gadE* might actually bring more burden to the strain.

The strains MG 1124, MG 0414, along with the wild type strain were then tested at the shake flask scale. The final OD_600_ of strains MG 1124 and MG 0414 were 2.96 and 3.20, which were 113% and 122% of the wild type strain (i.e., 2.62), and the maximum specific growth rates were 0.80 h^−1^ and 0.77 h^−1^, which were 93% and 90% of that of the wild type strain (i.e., 0.86 h^−1^). Thus, the two modified strains maintained advantages, albeit of reduced extents, over the wild type in terms of cell growth, whereas the maximum specific growth rates were comparable to that of the wild type strain.

### Biochemical analyses of the synthetic acid-tolerance modules

In order to shed some light over the acid tolerance mechanism, we determined the intracellular ROS levels in strains MG 1124, MG 0414 (i.e., the best strains of the four-gene and the three-gene module libraries in terms of OD_600_, respectively) and the wild type at pH 5.0 by a DCFH-DA-based fluorescent assay [50]. The results showed that in the wild type strain the ROS level increased rapidly during the first 4 h, decreased gradually until 12 h and then remained at a stable level (Additional file 1: Fig. S3). Differently, strains MG 1124 and MG 0414, showed low ROS levels through 2–24 h, indicating that the overexpression of the enzymes SodB and KatE did effectively scavenge intracellular ROS.

In addition, we examined the releases of GABA and ammonia in the strains during the growth at the shake flask scale (Additional file 2: Table S3). At the exponential phase (3 h), the releases of GABA in MG 1124 and MG 0414 were 18.9 μM/min and 12.8 μM/min, corresponding to 3.0- and 2.1-fold that of the wild type strain (i.e., 6.2 μM/min), respectively. At the stationary phase (16 h), the releases of GABA in MG 1124, MG 0414 and the wild type decreased to 1.7 μM/min, 7.9 μM/min, and 0.4 μM/min, respectively. The increased GABA release in strain MG 1124 can be rationalized as the consequence of the overexpression *gadE*. On the other hand, in strain MG 0414, in which *gadE* was not overexpressed, the release of GABA also increased, albeit to a lesser extent, thus additional mechanisms might contribute to the increased production of GABA other than the overexpression *gadE*. The rate of ammonia release in MG 1124 and MG 0414 at the exponential phase (3 h) were 0.5 μM/min and 0.4 μM/min, respectively, essentially equivalent to that of the wild type strain (0.5 μM/min). At the stationary phase (16 h) the rates of ammonia release in MG 1124 and MG 0414 were 6.7 μM/min and 10.2 μM/min, which were 48% and 73% of that at wild type strain (13.9 μM/min). These results indicated that in our design the expression of *ybaS* was not involved in the mechanism of enhanced acid tolerance.

### Application of the synthetic acid-tolerance modules for the production of lysine at low pH using 10-mL micro-bioreactors and 1.3-L parallel bioreactors

In order to evaluate whether the synthetic acid-tolerance modules can enhance the productivity of industrial *E. coli*, we tested the modules in the lysine-producing strain *E. coli* MG1655 SCEcL3. A series of 33 acid-tolerance modules were selected from the *gadE-hdeB*-*sodB*-*katE* and the *hdeB*-*sodB*-*katE* libraries, using a final OD_600_ > 120% of that of the wild type or a maximum specific growth rate > 100% of that of the wild type as selection criteria. The production robustness was first evaluated using 10-mL micro-bioreactors (Micro-Matrix), then the four best strains were further evaluated in 1.3-L parallel bioreactors (T&J-Minibox). The medium used was an industrial medium provided by China Oil & Foodstuffs Corporation (COFCO) (see “Methods and materials”). The initial pH of the fermentation medium was pH 6.8, but during fermentation it would drop to about 5.0, unless continuously adjusted to neutrality by adding a base (commonly aqueous ammonia) [51]. The test fermentation pH was set at 6.0 since preliminary experiments indicated that at lower pH values (i.e., pH 5.5 and pH 5) none of the strains could produce lysine titers and yields from glucose comparable to those obtained with the parent strain at neutral pH. The four acid-responsive promoters were therefore tested at pH 6.0 (Additional file 1: Fig. S4). The relative strengths resulted 91%, 88%, 52% and 22% of the wild type *asr* promoter at pH 5.0, and their pH response ratio between pH 6.0 and pH 7.0 were 1.63, 1.60, 1.23 and 1.91, respectively. These demonstrated that the promoters retained acid-responsive capability at the mild acidic pH of 6.0.

**Fig. 4:**
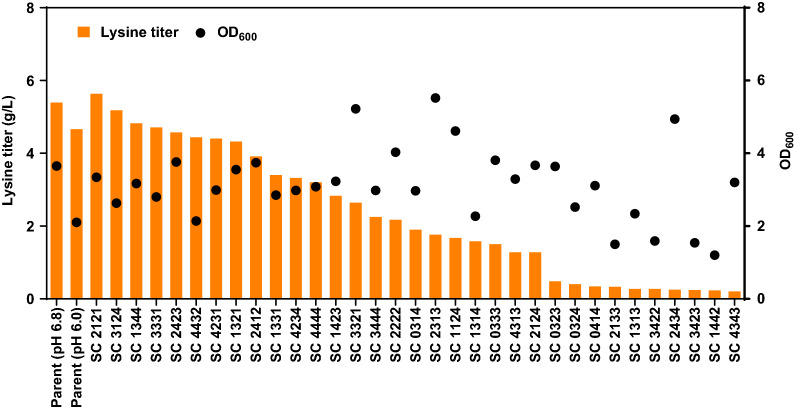
Influence of synthetic acid-tolerance modules on lysine titer in Micro-Matrix fermentations. The OD_600_ (black dots) and lysine titers (orange bars) of *E. coli* MG1655 SCEcL3 harboring different synthetic acid-tolerance modules and the parent strain *E. coli* MG1655 SCEcL3 cultured for 48 h in an industrial fermentation medium maintained at pH 6.8 or pH 6.0. Lysine titer and OD_600_ were measured at 48 h

The parent strain SCEcL3 was fermented at pH 6.0 or pH 6.8 for 48 h, while the other strains carrying the synthetic modules were fermented at pH 6.0 for 48 h. As shown in Fig. 4, in the Micro-Matrix format, the lysine titer of the parent strain was 4.7 g/L at pH 6.0, corresponding to 87% of that at pH 6.8 (i.e., 5.4 g/L), and the yield at pH 6.0 was 0.43 g/g glucose, corresponding to 78% of that at pH 6.8 (i.e., 0.55 g/g); the final OD_600_ was 2.1 at pH 6.0, corresponding to 58% of that at pH 6.8 (i.e., 3.7). For strains harboring acid-tolerance modules, the lysine titers of the four best strains SC 2121, SC 3124, SC 1344 and SC 3331 were 5.6 g/L, 5.4 g/L, 4.8 g/L and 4.7 g/L, which were 121%, 111%, 103% and 101% of that for the parent strain at pH 6.0 (i.e., 4.7 g/L), respectively. It is worth to note that the titers of SC 2121 and SC 3124 at pH 6 were comparable or higher than that of the parent strain at pH 6.8 (i.e., 5.4 g/L), indicating that the synthetic acid-tolerance modules could enhance the robustness of lysine production at pH 6.0. In addition, the lysine yields of the same four best strains were 110%, 113%, 90% and 111% of that of the parent strain at pH 6.0 (i.e., 0.43 g/g glucose), or 86%, 89%, 70% and 87% of that at pH 6.8 (i.e., 0.55 g/g glucose), respectively. The final OD_600_ of the four strains were all higher than that of the parent strain at pH 6.0 (i.e., 2.1), or 92%, 72%, 89% and 77% of that for the parent strain at pH 6.8 (i.e., 3.7), respectively.

**Fig. 5:**
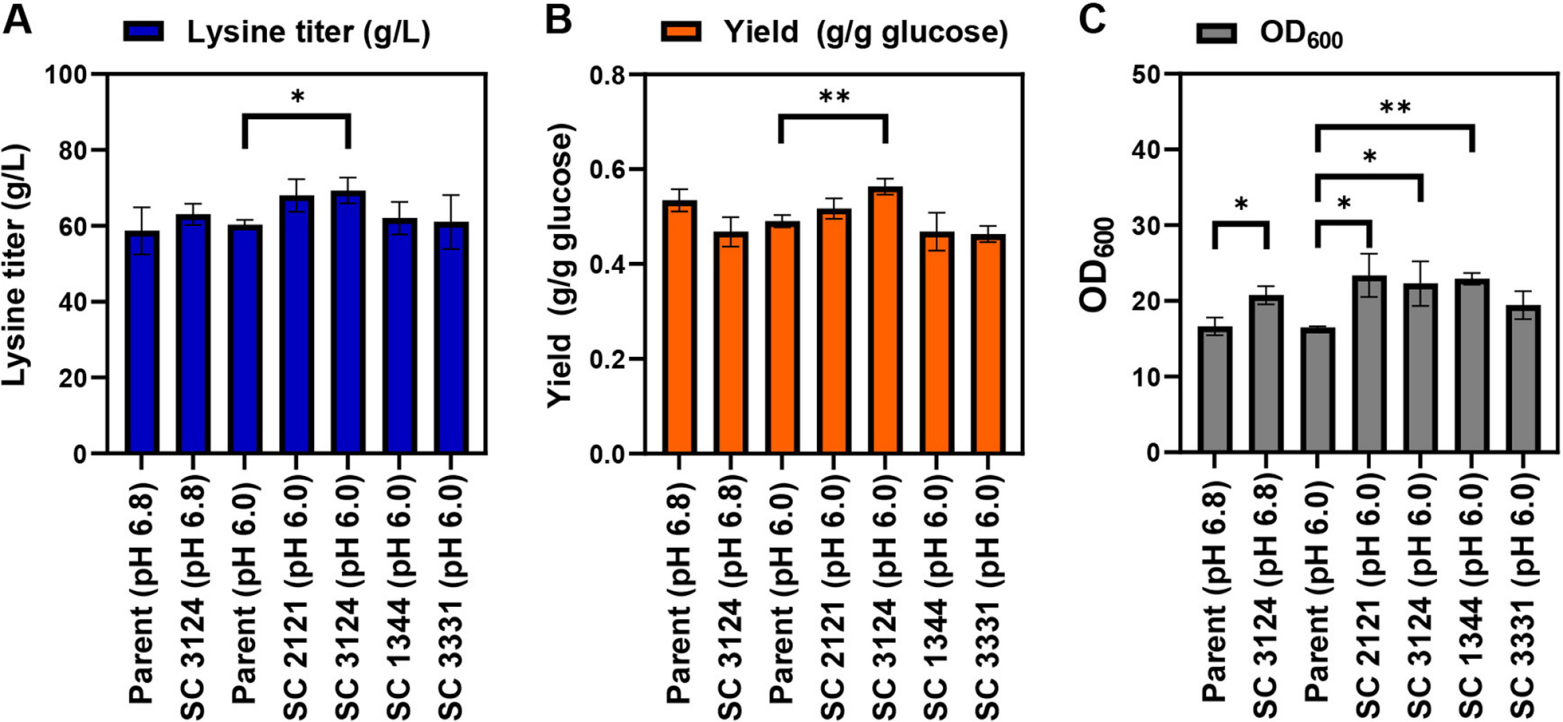
Influence of synthetic acid-tolerance modules on lysine production in 1.3-L parallel bioreactors. The lysine titers (**A**), lysine yields (**B**) and OD_600_ (**C**) of *E. coli* MG1655 SCEcL3 harboring different synthetic acid-tolerance modules and the parent strain *E. coli* MG1655 SCEcL3 cultured for 48 h in an industrial fermentation medium maintained at pH 6.8 or pH 6.0. The graph shows the results at 48 h. Each experiment was performed in three biological replicates. * Represents significance (p < 0.05). ** represents significance (p < 0.01)

We then further evaluated the same four strains in 1.3-L parallel bioreactors. Compared to the parent strain, the lysine titers of SC 2121, SC 3124, SC 1344 and SC3331 were 68.0 g/L, 69.3 g/L, 62.0 g/L and 61.0 g/L, which were 113%, 115%, 103% and 101% of that of the parent strain at pH 6.0 (i.e., 60.3 g/L), or 116%, 118%, 106% and 104% of that of the parent strain at pH 6.8 (i.e., 58.7 g/L), respectively (Fig. 5A). In addition, compared to the parent strain, the lysine yields of the four strains were 0.52 g/g, 0.56 g/g, 0.47 g/g and 0.46 g/g, which were 105%, 115%, 96%, 94% of that of the parent strain at pH 6.0 (i.e., 0.49 g/g glucose), or 97%, 105%, 88% and 87% of that for the parent strain at pH 6.8 (0.53 g/g), respectively (Fig. 5B). The final OD_600_ of the four strains were 23.4, 22.3, 22.9 and 19.5, which were 142%, 135%, 139% and 118% of that of the parent strain at pH 6.0 (i.e., 16.5), or 148%, 135%, 139% and 118% of that for the parent strain at pH 6.8 (i.e., 15.8), respectively (Fig. 5C). Thus, at pH 6.0, SC 3124 performed best in terms of the lysine titer, the yield, as well as the final OD_600_ (p < 0.05, FDR corrected), and with the lysine titer and yield comparable to those of the parent strain at pH 6.8. Moreover, we also evaluated SC 3124 at pH 6.8, and compared to the parent strain, only the difference in biomass was significant (p < 0.05, FDR corrected), suggesting that the synthetic acid-tolerance module improved the performance of the best strain by mostly enhancing the growth robustness.

## Discussion

During industrial bioprocesses, the gradual decrease of environmental pH causes multiple stresses that impair cell growth and lower the fermentation yields [7, 52–55]. Engineering acid resistant strains is potentially an important strategy to increase productivity, decrease base consumption, and facilitate the downstream separations [7]. Synthetic biology is an inspiring route to construct acid-tolerance modules that are functional in industrial strains. However, it is difficult to assemble reasonable stress-tolerance modules from hundreds of stress-tolerant genes. For example, in *E. coli*, there are at least 274 genes involved in acid stress regulation [12], and 217 genes involved in oxidative stress regulation [56]. Based on our previous observations [15, 25], in this study, we focused our attention on the proton-consuming acid resistance system AR2, periplasmic chaperones, and ROS scavengers. We successfully obtained two improved lysine-producing *E. coli* strains, namely SC 3124 and SC 2121, for which the lysine titers at pH 6.0 were 118% and 116% of that of the parent strain at pH 6.8, and the lysine yields from glucose were 105% and 97% of that of the parent strain at pH 6.8. In addition, the lysine yield for SC 3124 was 115% of the parent strain at pH 6.0. By a rough estimation, this yield could save about 260 kg of glucose per ton of lysine produced, while fermentation at pH 6.0 could save 5–10% of the amount of acid added during purification of lysine in industrial settings.

This study strengthens the notion that periplasmic molecular chaperons are important for acid resistance of *E. coli* [15, 33]. This is likely because when the protons from the acidic environment permeates into the cell, the periplasmic space becomes the first line of defense and the periplasmic proteins become vulnerable, thus requiring the protection afforded by chaperones [26, 33]. Although the glutathione (GSH) system has been used for ROS scavenging to increase the tolerance for bacterial or yeast cells, it requires a complex engineering route to balance the NADPH/NADP^+^ ratio [28, 57]. Thus, an enzymatic system like SodB-KatE [44] rather than GSH might be more advantageous.

Along this line, it is interesting to note that at the cell growth assay level, we found that *gadE* was not a necessary component for the synthetic acid tolerance modules, but at the lysine productivity level, the best strains all contained synthetic modules with this gene (in addition to its genomic copy). As GadE is responsible for regulating the AR2 system, we attempted to replace it with the two AR2 component genes coding for GadB (variant GadB(dHT) which has enhanced activity at pH 4–6 [58]), and YbaS, but the effort only yielded strains with lower performance both in terms of cell growth and lysine productivity (data not shown). There is a possibility that overexpressing *gadE* could up-regulate the lysine-dependent acid resistance system (AR4) [30]. This remain to be further investigated.

Recently, a combinatorial strategy was used for engineering multiple-stress-tolerant ethanol-producing *Saccharomyces cerevisiae* strains [44]. The study involved a much larger library of four sets of genes, more specifically, a set of 5 genes coding for heat shock proteins with chaperone functions, 16 genes involved in the antioxidant system, 5 genes coding for ubiquitins and autophagy proteins that remove denatured proteins, and 6 transcription factors that regulate stress-related networks were combined via the golden gate assembly with 14 different promoters and screened at high temperatures (35 °C or 37 °C). The best strain obtained in terms of ethanol titer was found to contain overexpressed *sodA*, *sodB* from *E. coli* and an endogenous cAMP phosphodiesterase *PDE2*. It appeared that *PDE2* alone activated a number of stress tolerance pathways, and thus the effects of other factors like the heat shock proteins, ubiquitins and autophagy proteins were likely masked under the overexpression of *PDE2* [59–61]. This study also hints that *PDE2* like regulators with an appropriate activation range could also be tested for *E. coli*. The best strain finally was subjected to adaptive evolution by atmospheric and room temperature plasmas (ARTP), yielding a final strain with an enhanced ethanol titer of 6.9% in a large scale 200-L fermenter.

In this work, we constructed a synthetic *asr* promoter library with an enlarged operation range both in terms of absolute promoter strength as well as of pH response ratio at different pH (pH 5.0 vs pH 7.0). This promoter is regulated by RstBA and PhoRB systems. The RstA box is located at − 77 to − 55 of the promoter region, and the PhoB is located at − 39 to − 22, thus overlaps the -35 element (Additional file 1: Fig. S1) [37, 38]. RstBA induces *asr* under mild acidic conditions, while PhoRB induces *asr* at low inorganic phosphate concentrations [38, 62]. Previous saturation mutagenesis experiments performed at the RstA box [63] produced only four notable variants with strengths at pH 5.0 ranging from 1.6 to 40.0% of the wild type, and the pH response ratios ranging from 0.7 to 14.4% of the wild type. Therefore, we chose to target the 9 bp spacer region between PhoB box and − 10 sequence, which has been shown to affect the strengths of several promoters [39, 40]. Our strategy proved to be successful, and the resulting series of variants may be useful for constructing other acid-responsive synthetic modules or circuits [8]. It is noteworthy that the mutations in this region also can cause loss of the acid responsiveness of the promoter, even though it is located downstream of the RstA box. However, a previous study on the engineering of an osmic stress-responsive *ect* promoter also showed that the mutations or deletions in the spacer region of the promoter changed the osmotic responsiveness [43].

## Conclusions

This study demonstrates a useful strategy to improve growth robustness and productivity for industrial *E. coli* in fermentation at mildly acidic pH, through the use of synthetic acid tolerance modules, which involve only a limited number of genes, in our case, periplasmic protein chaperone (*hdeB*), ROS scavenging enzymes (*sodB* and *katE*), and a transcription factor for the AR system (*gadE*). The functional optimization of these modules can be realized by the use of acid-responsive promoters of different strengths, and screened with a stepwise protocol, from a growth-guided high-throughput screening to a production performance-guided fermentation evaluation.

## Methods and materials

### Strains, plasmids, and materials

The strains and plasmids used in this study are listed in Additional file 2: Table S4. The DNA sequences of the primers used in this study are listed in Additional file 2: Table S5, and genes used in this study are listed in Additional file 2: Table S6. Restriction enzymes, Q5 DNA polymerase and T4 DNA ligase were purchased from New England Biolabs (Beverly, MA, USA). pMD™19T Vector Cloning Kit was purchased from TakaRa (Dalian, China). Oligonucleotides synthesis and sequence analysis were performed by Sangon Biotech (Shanghai, China). The kits for DNA purification, gel recovery, genomic DNA extraction and plasmid mini-prep were purchased from Tiangen (Beijing, China). The 96 well black flat clear bottom polystyrene microplates were purchased from Corning (New York, USA). All chemicals were purchased from Sigma-Aldrich (Shanghai, China) or Sangon Biotech (Shanghai, China).

### Culture media

For cloning, all *E. coli* MG1655 strains were cultured in lysogeny broth (LB) medium at 37 °C [50]. All *E. coli* MG1655 strains were tested in LB supplemented with 20 g/L glucose (LBG medium) for the cell growth assay. For fermentation of *E. coli* MG1655 SCEcL3 strains, the seed medium consisted of sucrose (3 g/L), yeast extract (5 g/L), tryptone (7 g/L), ammonium sulfate (5 g/L), potassium dihydrogen phosphate (5 g/L), magnesium sulfate (0.5 g/L), ferrous sulphate (0.012 g/L), manganous sulfate (0.012 g/L), sodium glutamate (5 g/L), l-threonine (0.3 g/L), l-methionine (0.3 g/L) and pyruvic acid (0.3 g/L), and the fermentation medium consisted of glucose (30 g/L), phosphoric acid (0.6 g/L), magnesium sulfate (2 g/L), ammonium sulfate (10 g/L), corn steep liquor (0.325 g/L), potassium chloride (0.5 g/L), betaine (2.2 g/L), ferrous sulphate (0.032 g/L), manganous sulfate (0.032 g/L), l-threonine (0.25 g/L), cupric sulfate (6.8 mg/L), zinc sulfate (7.65 mg/L), and thiamine (5.6 mg/L). For the strains harboring plasmids with chloramphenicol or ampicillin resistance, 34 μg/mL chloramphenicol or 50 μg/mL ampicillin was added to the medium.

### Promoter library construction and screening

The synthetic *asr* promoter library was constructed by the Gibson assembly [64], based on plasmid pAm that was constructed in house based using plasmid pACYC184 as the backbone. Two assembling fragments were PCR-amplified by two pairs of primers Pasrlib-mCherrygi1-F/Pasrlib-mCherrygi1-R (containing the randomly mutated region of the promoter), and Pasrlib-mCherrygi2-F/Pasrlib-mCherrygi2-R from plasmid pAm. After purification, the two fragments were assembled and transformed by electroporation into *E. coli* XL1-Blue. Transformed cells were cultivated on LB agar plates overnight at 37 °C. The library size was estimated to be 10^5^. Plasmids were then extracted and transformed by electroporation into *E. coli* MG1655 for screening.

For the screening of the *asr* promoter variants, the cells were grown overnight (about 16 h) in LBG medium (pH 7.0) at 37 °C. Then the cultures were diluted to initial OD_600_ 0.05 in 200 μL LBG medium (pH 7.0) or LBG medium (pH 5.0) which was acidified by HCl in 96-well black flat clear bottom polystyrene microplates. The cultures were grown at 37 °C for 16 h, and OD_600_ and fluorescence of mCherry (λEx: 553 nm, λEm: 583 nm) were determined by a Tecan infinite 200 pro microplate reader (Tecan Group Ltd., Männedorf, Switzerland). The clones with a fold of the fluorescence/OD_600_ ratio between pH 5.0 and pH 7.0, i.e. pH response ratio, above 1.8 were selected to perform the second screening to verify the performance. Each experiment was performed in three biological replicates. Sequences of the mutated region at *asr* promoter variants in this study are available in Additional file 2: Table S7.

### Construction of recombinant strains library with acid-tolerance modules

For evaluating *gadE*, *hdeB*, *sodB* and *katE* individually, the plasmids (total 12 plasmids) containing individual genes with three P*asr* variants (P24E8, P77C4 and P50G1, these were numbered 1, 3, 4, respectively, also see below) and the rrnB terminator were constructed using the Gibson assembly based on the pAm plasmid series containing different P*asr* variants (Fig. 2A). Take the construction of *gadE*-containing plasmids pAg1, pAg3 and pAg4 as examples, the *gadE* gene fragment with two homologous arms was PCR-amplified from *E. coli* MG1655 genomic DNA by a pair of primers gadEgi-F/gadEgi-R, and the other two assembling fragments were PCR-amplificated by two pairs of primers Pasr-comgi1-F/Pasr-mCherrygi1-R and Pasr-comgi2-F/Pasr-comgi2-R from pAm1, pAm3 and pAm4. These three sets of fragments were then assembled to generate plasmids pAg1, pAg3 and pAg4, respectively. The plasmids (total three plasmids) overexpressing the *katE*-*sodB* cassette were constructed based on plasmids pAk1, pAk3 and pAk4 containing *katE* in a similar fashion (Fig. 2A).

The *gadE*-*hdeB*-*sodB*-*katE* and *hdeB*-*sodB*-*katE* acid-tolerance modules were constructed using Golden Gate assembly [65]. First, a fourth promoter P10G6 was introduced, and this promoter was numbered 2, in addition to P24E8, P77C4 and P50G1, a new set of plasmids containing the four individual genes placed under the control of this promoter, pAg2, pAh2, pAs2 and pAk2 were then constructed using the aforementioned Gibson assembly method. Then a series of pMD19T-derived plasmids (total 16 plasmids) were constructed for use in the Golden Gate assembly. Take the construction of *gadE*-containing plasmids pMg1, pMg2, pMg3 and pMg4 for examples, the fragments of *gadE* with P*asr* variants (P24E8, P10G6, P77C4 and P50G1) and the rrnB terminator were PCR-amplificated by a pair of primers BsaI-f1-f2-F/BsaI-f1-f2-R with *Bsa*I restriction sites from plasmids pAg1, pAg2, pAg3 and pAg4, respectively. Then the fragments were inserted into the pMD19T vector using the pMD™19T Vector Cloning Kit, to generate plasmids pMg1, pMg2, pMg3 and pMg4. For the *gadE*-*hdeB*-*sodB*-*katE* acid-tolerance modules, all the pMD19T-derived plasmids (total 16 plasmids) and the destination plasmid pAcc1 (constructed in house based on plasmid pACYC184) were assembled using the Golden Gate assembly. For the *hdeB*-*sodB*-*katE* acid-tolerance modules, all the pMD19T-derived plasmids except for pMg1, pMg2, pMg3 and pMg4 (total 12 plasmids) and the destination plasmid pAcc2 (constructed in house based on plasmid pACYC184) were assembled. Then the plasmids were transformed by electroporation into *E. coli* MG1655, and the transformed cells were cultivated on LB agar plates overnight at 37 °C.

### Cell growth assay

The cell growth assays on were performed using a modification of the protocol as described in our pervious study [25]. Wild type *E. coli* MG1655 and strains harboring acid-tolerance modules were grown overnight (about 16 h) in LBG medium (pH 7.0) at 37 °C, and then the cultures were diluted to initial OD_600_ 0.05 in 300 μL LBG medium (pH 5.0) which was acidified by HCl. Then the cultures were incubated at 37 °C in 100-well Honeycomb microplates monitor by automated turbidimeter (Bioscreen C, Oy Growth Curves Ab Ltd., Helsinki, Finland) for online monitoring of OD_600_ for 24 h. Each experiment was performed in three biological replicates.

### Statistics analysis

The Analysis of Variance (ANOVA) was performed using Design Expert software version 10 for Windows (Stat-Ease Inc.). The ANOVA F-statistics test and the general linear regression model were used [49]. Briefly, the linear regression model of four factorial ANOVA can be described as:

$${y}_{ijklr}=\mu +{\alpha }_{i}+{\beta }_{j}+{\gamma }_{k}+{\delta }_{l}+{(\alpha \beta )}_{ij}+{(\alpha \gamma )}_{ik}+{(\alpha \delta )}_{il}+{(\beta \gamma )}_{jk}+{(\beta \delta )}_{jl}+{(\gamma \delta )}_{kl}+{(\alpha \beta \gamma )}_{ijk}+{(\alpha \beta \delta )}_{ijl}+{(\alpha \gamma \delta )}_{ikl}+{(\beta \gamma \delta )}_{jkl}+{(\alpha \beta \gamma \delta )}_{ijkl}+\varepsilon$$

where *μ* is the population mean, *α*_*i*_, *β*_*j*_, *γ*_*k*_ and *δ*_*l*_ are main effects of four factors due to the ith, jth, kth and lth level of the factors, *ε* is the random error, and the other parameters accounting for the interactions among the factors. In this study, *α*, *β*, *γ* and *δ* denoted the genes *gadE*, *hdeB*, *sodB* and *katE* as the four factors, and the levels of the factors were the four *asr* promoter variants of different strengths, P24E8, P10G6, P77C4 and P50G1.

Statistical significance analysis was performed using the two-tailed Student’s t-test with a confidence interval of 95%, and p-value (which was corrected by false-discovery rate (FDR)) below 0.05 indicates significance.

### Selection of lysine producing strains harboring synthetic acid-tolerance modules by 10-mL micro-bioreactor (Micro-Matrix) fermentation

The lysine producing parent strain *E. coli* MG1655 SCEcL3 was provided by Nutrition and Health Research Institute (Beijing, China), a unit of COFCO. *E. coli* MG1655 SCEcL3 and SCEcL3 harboring synthetic acid-tolerance modules were grown overnight at 37 °C in seed medium. The overnight cultures were diluted (1.5:10) into 24-well Micro-Matrix plates containing 3.6 mL fermentation medium (pH 7.0, adjusted by 25% (w/v) ammonia) in Micro-Matrix (Applikon Biotechnology, Heertjeslaan, Netherland). The fermentation was performed at 37 °C, 300 rpm for 48 h. The dissolved oxygen (DO), pH and temperature were monitored online. The initial pH of the fermentation medium for all the strains was pH 6.8, and after the pH gradually decreased during fermentation, the wild type strains were controlled at pH 6.8 or pH 6.0, while other strains were controlled at pH 6.0, using 5% (w/v) ammonia. DO and temperature were controlled at 40–60%, and 37.0 ± 0.1 °C, respectively, during the 48 h fermentation period. After 48 h fermentation, samples were taken for measuring optical density, residual glucose and lysine titer. OD_600_ was measured by ultraviolet spectrophotometer and the titers of glucose and lysine were measured by SBA-40D biosensor analyzer (Shandong Academy of Science, Shandong Province, China) [66].

### Lysine fermentation at parallel bioreactors

Fermentation test was performed in T&J-Minibox 1.3-L × 8 parallel bioreactors (T&J Bio-engineering Co., Ltd., Shanghai, China). Briefly, cells were activated in seed medium and inoculated at 1% (v/v) into 500 mL flasks containing 100 mL seed medium at 37 °C, 250 rpm until the OD_600_ reaching 2.0–3.0, and 75 mL seed inoculum was then inoculated into 425 mL fermentation medium in the 1.3-L parallel bioreactors. The DO, pH and temperature were monitored online. The pH of the cultures was controlled at 6.8 or 6.0 by the addition of 5% (w/v) ammonia. DO, temperature, and glucose were controlled at 40–60%, 37.0 ± 0.1 °C, and 0.4–1.0% (w/v), respectively, during the 48 h fermentation period. DO was monitored online and was controlled by adjusting the agitation rate from 200 to 1000 rpm with an aeration rate of 0.5 vvm. Samples were taken periodically for measuring optical density, residual glucose and lysine. Each experiment was performed in three biological replicates.

### GABA and ammonia release assays

The assays of GABA and ammonia release were followed as previously described with minor modifications [15]. For the GABA release assay, cultures grown in LBG medium were harvested in exponential phase (3 h) or in stationary phase (16 h). The pellet was washed twice with citrate buffer (25 mM, pH 5.0) and resuspended to 1 OD_600_/mL with 1 mL citrate buffer (25 mM, pH 5.0, containing 10 mM l-glutamate). After incubation at 37 °C for 1 h, the cultures were harvested by centrifugation and the supernatant was used for subsequent analysis. Briefly, in a 100 μL reaction mixture, 10 μL of supernatant was mixed with 0.5 mM NADP^+^, 0.5 mM dithiothreitol, 0.5 mM α-ketoglutarate and 10 μg GABase (Sigma-Aldrich, Shanghai, China). The reaction mixture was incubated at 37 °C for 1 h, and then measured at 340 nm using a Tecan infinite 200 pro microplate reader (Tecan Group Ltd., Männedorf, Switzerland). The concentration of GABA was calculated using a standard curve obtained with a set of GABA standard solutions of different concentrations.

For the ammonia release determination assay, cultures grown in LBG medium were harvested in exponential phase (3 h) or in stationary phase (16 h). The pellet was washed twice with citrate buffer (25 mM, pH 5.0) and resuspended to 1 OD_600_/mL with 20 mL citrate buffer (25 mM, pH 5.0, containing 10 mM l-glutamine). The concentration of ammonium ions was continuously monitor using a SevenCompac™ S220 electrochemical analytical meter (Mettler-Toledo AG, Schwerzenbach, Switzerland) with an ammonia ion selective electrode (model DX218-NH4+, Mettler-Toledo GmbH, Schwerzenbach, Switerland).

### Measurement of intracellular ROS level

The 2ʹ,7ʹ-dichlorofluorescein diacetate (DCFH-DA) based assay for measurement of the intracellular ROS level was followed as previously described [36]. The samples were prepared as follows. Wild type *E. coli* MG1655, MG 1124 and MG 0414 were grown overnight (16 h) in LBG medium (pH 7.0) at 37 °C, and then the cultures were diluted to initial OD_600_ 0.05 in 250 mL LBG medium (pH 5.0) which was acidified by HCl in shake flasks. Then the cultures were incubated at 37 °C, 250 rpm. The cultures of different time points (2 h, 4 h, 8 h, 12 h and 24 h) were harvested and washed twice with 50 mM PBS (pH 7.4).

## Supplementary Information

**Additional file 1: Figure S1.** Schematic diagram of the construction of the synthetic *asr* promoter library. **Figure S2.** Distribution of 177 unique *asr* promoter variants in terms of fluoresce/OD_600_ at pH 5.0 and 7.0. **Figure S3.** Statistics of nucleotide frequency in N9 sequences of two groups of *asr* promoter variants with the pH response ratios above 1.8. **Figure S4.** Intracellular ROS level in strains MG 1124, MG 0414 and *E. coli* MG1655 (WT) cultured for 24 h in LBG at pH 5.0. **Figure S5.** Relative strength of the synthetic *asr* promoter library entries at different pH.

**Additional file 2: Table S1.** Summary of the relative strengths of the *asr* promoter library entries at pH 5.0. **Table S2.** Multi-factorial ANOVA analysis of the recombinant strains in *gadE*-*hdeB*-*sodB*-*katE* library. **Table S3.** The release of GABA and ammonia in the exponential phase and in the stationary phase. **Table S4.** Strains and plasmids used in this study. **Table S5.** Primers used in this study. **Table S6.** Sequence of DNA fragments or genes used in this study. **Table S7.** Sequences of the mutated region at *asr* promoter variants in this study with relative strength at pH 5.0 and pH response ratio.

**Additional file 3.** Growth characteristics (final OD_600_ ratio and maximum specific growth rate ratio) of the strains containing the *gadE*-*hdeB*-*sodB*-*katE* acid-tolerance modules.

**Additional file 4.** Growth characteristics (final OD_600_ ratio and maximum specific growth rate ratio) of the strains containing the *hdeB*-*sodB*-*katE* acid-tolerance modules.

## Data Availability

All data generated or analyzed during this study are included in this published article and its additional files.

## Abbreviations

ALE: Adaptive laboratory evolution
ANOVA: Analysis of variance
AR: Acid resistance
AR2: Acid resistance system 2
ARTP: Atmospheric and room temperature plasmas
*asr*: Acid shock RNA
COFCO: China Oil & Foodstuffs Corporation
CREATE: CRISPR enabled trackable genome engineering
CRISPR: Clustered regularly interspaced short palindromic repeats
DCFH-DA: 2ʹ,7ʹ-Dichlorofluorescein diacetate
DO: Dissolved oxygen
*E. coli*: *Escherichia coli*
EP: Exponential phase
GABA: γ-Amino butyric acid
GDP: Gross domestic product
gTME: Global transcription machinery engineering
OD_600_: Optical density at 600 nm
ROS: Reactive oxygen species
SCRaMbLE: Synthetic chromosome rearrangement and modification by loxP-mediated evolution
SP: Stationary phase
sRNA: Small RNA
