# Supplementary material for: Synthetic acid stress-tolerance modules improve growth robustness and lysine productivity of industrial Escherichia coli in fermentation at low pH
Source: Microb Cell Fact. 2022 Apr 22;21:68. doi: 10.1186/s12934-022-01795-4 (PMC9026648; doi:10.1186/s12934-022-01795-4)
Supplement: Supplementary file 1 — Additional file 1: Figure S1. Schematic diagram of the construction of the synthetic asr promoter library. Figure S2. Distribution of 177 unique asr promoter variants in terms of fluoresce/OD600 at pH 5.0 and 7.0. Figure S3. Statistics of nucleotide frequency in N9 sequences of two groups of asr promoter variants with the pH response ratios above 1.8. Figure S4. Intracellular ROS level in strains MG 1124, MG 0414 and E. coli MG1655 (WT) cultured for 24 h in LBG at pH 5.0. Figure S5. Relative strength of the synthetic asr promoter library entries at different pH. [file 12934_2022_1795_MOESM1_ESM.docx]

Additional file 1

Figures S1-S5


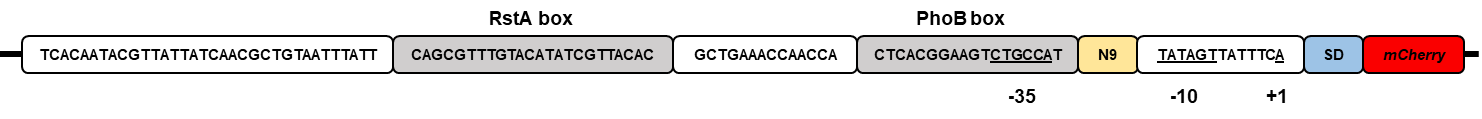


**Fig. S1** Schematic diagram of the construction of the synthetic *asr* promoter library. N9 represents the 9 bp spacer sequence between PhoB box and -10 sequence of the wild type *asr* promoter that was randomized. RstA box and PhoB box are represented in gray; Underlined nucleotides represent the -35, -10 and transcription start site (+1) sequences; SD represents the Shine-Dalgarno sequence; mCherry represents the red fluorescence protein gene sequence.

**Fig. S2** Distribution of 177 unique *asr* promoter variants in terms of fluoresce/OD_600_ at pH 5.0 and 7.0. The orange dots represent the clones with *asr* promoter variant whose pH response ratio are above 1.8. The black dots represent *asr* promoter variant whose pH response ratio are below 1.8. The blue dot represents wild type *asr* promoter. The black dash line represents the pH response ratio = 1.0. The red solid line represents the pH response ratio = 1.8.

**Fig.** **S3** Statistics of nucleotide frequency in N9 sequences of two groups of *asr* promoter variants with the pH response ratios above 1.8. The original nucleotides of the wild type *asr* promoter are shown below the graph.

**Fig.** **S4** Intracellular ROS level in strains MG 1124, MG 0414 and *E. coli* MG1655 (WT) cultured for 24 h in LBG at pH 5.0. The DCFH-DA based fluorescence was measured every 30 min. Each experiment was performed in three biological replicates.


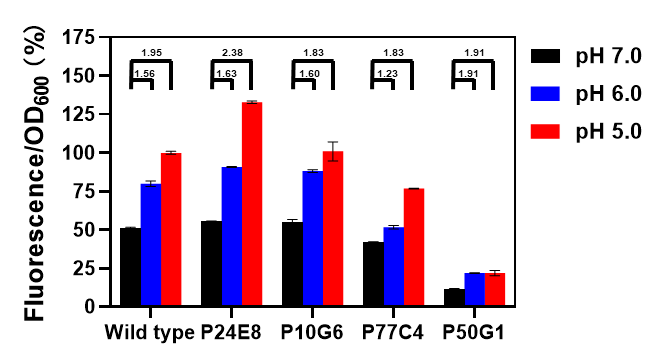


**Fig.** **S5** Relative strength of the synthetic *asr* promoter library entries at different pH. The relative strength was defined as the strength of each promoter variants compared to the wild type *asr* promoter determined by cell growth assay after fermentation in LGB for 16 h at pH 7.0 (black), pH 6.0 (blue) and pH 5.0 (red). The number above the columns represent the pH response ratio. Each experiment was performed in three biological replicates.
