# Supplementary material for: Synthetic acid stress-tolerance modules improve growth robustness and lysine productivity of industrial Escherichia coli in fermentation at low pH
Source: Microb Cell Fact. 2022 Apr 22;21:68. doi: 10.1186/s12934-022-01795-4 (PMC9026648; doi:10.1186/s12934-022-01795-4)
Supplement: Supplementary file 2 — Additional file 2: Table S1. Summary of the relative strengths of the asr promoter library entries at pH 5.0. Table S2. Multi-factorial ANOVA analysis of the recombinant strains in gadE-hdeB-sodB-katE library. Table S3. The release of GABA and ammonia in the exponential phase and in the stationary phase. Table S4. Strains and plasmids used in this study. Table S5. Primers used in this study. Table S6. Sequence of DNA fragments or genes used in this study. Table S7. Sequences of the mutated region at asr promoter variants in this study with relative strength at pH 5.0 and pH response ratio. [file 12934_2022_1795_MOESM2_ESM.docx]

Additional file 2

Tables S1-S7

**Table S1** Summary of the relative strengths of the *asr* promoter library entries at pH 5.0.

| Relative strength range | Amount | Description（versus wild type P*asr*） |
| --- | --- | --- |
| 110-130% | 4 | 133%，126%，123%，119% |
| 90-110% | 16 | 108%，108%，106%，106%，105%，105%，105%，102%，102%，102%，101%，98%，96%，95%，93%，90% |
| 70-90% | 13 | 88%，87%，87%，83%，82%，81%，79%，78%，78%，77%，75%，72%，70% |
| 50-70% | 6 | 67%，66%，61%，61%，60%，57% |
| 30-50% | 4 | 46%，38%，36%，34% |
| 10-30% | 6 | 29%，29%，27%，25%，23%，22% |

**Table S2** Multi-factorial ANOVA analysis of the recombinant strains in *gadE*-*hdeB*-*sodB*-*katE* library.

| Source | Sum of squares | Mean square | F value | p value |
| --- | --- | --- | --- | --- |
| Model | 3255.240 | 217.020 | 2.070 | 0.012^1^ |
| *gadE* | 3.160 | 3.160 | 0.030 | 0.862 |
| *hdeB* | 749.170 | 749.170 | 7.150 | 0.008^1^ |
| *sodB* | 1111.310 | 1111.310 | 10.61 | 0.001^1^ |
| *katE* | 387.190 | 387.190 | 3.700 | 0.056 |
| *gadE* × *hdeB* | 0.052 | 0.052 | 4.920E-4 | 0.982 |
| *gadE* × *sodB* | 88.570 | 88.570 | 0.850 | 0.359 |
| *gadE* × *katE* | 245.650 | 245.650 | 2.350 | 0.127 |
| *hdeB* × *sodB* | 134.260 | 134.260 | 1.280 | 0.259 |
| *hdeB* × *katE* | 60.430 | 60.430 | 0.580 | 0.448 |
| *sodB* × *katE* | 13.790 | 13.790 | 0.130 | 0.717 |
| *gadE* × *hdeB* × *sodB* | 6.350 | 6.350 | 0.061 | 0.806 |
| *gadE* × *hdeB* × *katE* | 60.050 | 60.050 | 0.570 | 0.450 |
| *gadE* × *sodB* × *katE* | 34.770 | 34.770 | 0.330 | 0.565 |
| *hdeB* × *sodB* × *katE* | 105.360 | 105.360 | 1.010 | 0.317 |
| *gadE* × *hdeB* × *sodB* × *katE* | 7.780 | 7.780 | 0.074 | 0.786 |
| Residual | 23877.850 | 104.730 |  |  |

^1^ p < 0.05 indicates statistical significance

**Table S3** The release of GABA and ammonia in the exponential phase and in the stationary phase.

| Strains | Rate in the exponential phase (μM/min) | Rate in the stationary phase (μM/min) |
| --- | --- | --- |
| The release of GABA | | |
| WT | 6.2 ± 1.7 | 0.4 ± 1.3 |
| MG 1124 | 18.9 ± 5.0 | 1.7 ± 0.4 |
| MG 0414 | 12.8 ± 2.1 | 7.9 ± 0.5 |
| The release of ammonia | | |
| WT | 0.5 ± 0.1 | 13.9 ± 5.1 |
| MG 1124 | 0.4 ± 0.1 | 6.7 ± 5.1 |
| MG 0414 | 0.5 ± 0.1 | 10.2 ± 8.9 |

**Table S4** Strains and plasmids used in this study.

| Strains or plasmids | Description | Source |
| --- | --- | --- |
| Strains | | |
| WT | Wild type *E. coli* MG1655 strain | Lab collection |
| P*asr*-*mCherry* | *E. coli* MG1655, pAm (pACYC184-Pasr-*mCherry*), Cm^R^ | Lab collection |
| P*asr* variants-*mCherry* | *E. coli* MG1655, pAmV (pACYC184-Pasr variants-*mCherry*), Cm^R^ | This study |
| P24E8-*gadE* | *E. coli* MG1655, pAg1 (pACYC184-P24E8-*gadE*), Cm^R^ | This study |
| P77C4-*gadE* | *E. coli* MG1655, pAg3 (pACYC184-P77C4-*gadE*), Cm^R^ | This study |
| P50G1-*gadE* | *E. coli* MG1655, pAg4 (pACYC184-P50G1-*gadE*), Cm^R^ | This study |
| P24E8-*hdeB* | *E. coli* MG1655, pAh1 (pACYC184-P24E8-*hdeB*), Cm^R^ | This study |
| P77C4-*hdeB* | *E. coli* MG1655, pAh3 (pACYC184-P77C4-*hdeB*), Cm^R^ | This study |
| P50G1-*hdeB* | *E. coli* MG1655, pAh4 (pACYC184-P50G1-*hdeB*), Cm^R^ | This study |
| P24E8-*sodB* | *E. coli* MG1655, pAs1 (pACYC184-P24E8-*sodB*), Cm^R^ | This study |
| P77C4-*sodB* | *E. coli* MG1655, pAs3 (pACYC184-P77C4-*sodB*), Cm^R^ | This study |
| P50G1-*sodB* | *E. coli* MG1655, pAs4 (pACYC184-P50G1-*sodB*), Cm^R^ | This study |
| P24E8-*katE* | *E. coli* MG1655, pAk1 (pACYC184-P24E8-*katE*), Cm^R^ | This study |
| P77C4-*katE* | *E. coli* MG1655, pAk3 (pACYC184-P77C4-*katE*), Cm^R^ | This study |
| P50G1-*katE* | *E. coli* MG1655, pAk4 (pACYC184-P50G1-*katE*), Cm^R^ | This study |
| P24E8-*katE*-*sodB* | *E. coli* MG1655, pAks1 (pACYC184-P24E8-*katE*-*sodB*), Cm^R^ | This study |
| P77C4-*katE*-*sodB* | *E. coli* MG1655, pAks3 (pACYC184-P77C4-*katE*-*sodB*), Cm^R^ | This study |
| P50G1-*katE*-*sodB* | *E. coli* MG1655, pAks4 (pACYC184-P50G1-*katE*-*sodB*), Cm^R^ | This study |
| MG 1124 | *E. coli* MG1655, 1124 (pACYC184-24E8-*gadE*-24E8-*hdeB*-10G6-*sodB*-50G1-*katE*), Cm^R^ | This study |
| MG 0414 | *E. coli* MG1655, 0414 (pACYC184-50G1-*hdeB*-24E8-*sodB*-50G1-*katE*), Cm^R^ | This study |
| *E. coli* MG1655 SCEcL3 | secret | Gift of China Oil and Foodstuffs Corporation |
| SC 2121 | *E. coli* MG1655 SCEcL3, 2121 (pACYC184-P10G6-*gadE*-P24E8-*hdeB*-P10G6-*sodB*-P24E8-*katE*), Cm^R^ | This study |
| SC 3124 | *E. coli* MG1655 SCEcL3, 3124 (pACYC184-77C4-*gadE*-24E8-*hdeB*-10G6-*sodB*-50G1-*katE*), Cm^R^ | This study |
| SC 1344 | *E. coli* MG1655 SCEcL3, 1344 (pACYC184-P24E8-*gadE*-P77C4-*hdeB*-P50G1-*sodB*-P50G1-*katE*), Cm^R^ | This study |
| SC 3331 | *E. coli* MG1655 SCEcL3, 3331 (pACYC184-P77C4-*gadE*-P77C4-*hdeB*-P77C4-*sodB*-P24E8-*katE*), Cm^R^ | This study |
| Plasmids | | |
| pAm | pACYC184, P*asr*-*mCherry*, Cm^R^ | Lab collection |
| pAm1 | pACYC184, P24E8-*mCherry*, Cm^R^ | This study |
| pAm2 | pACYC184, P77C4-*mCherry*, Cm^R^ | This study |
| pAm3 | pACYC184, P50G1-*mCherry*, Cm^R^ | This study |
| pAm4 | pACYC184, P10G6-*mCherry*, Cm^R^ | This study |
| pAmV | pACYC184, P*asr* variants-*mCherry*, Cm^R^ | This study |
| pAg1 | pACYC184, P24E8-*gadE*, Cm^R^ | This study |
| pAg2 | pACYC184, P10G6-*gadE*, Cm^R^ | This study |
| pAg3 | pACYC184, P77C4-*gadE*, Cm^R^ | This study |
| pAg4 | pACYC184, P50G1-*gadE*, Cm^R^ | This study |
| pAh1 | pACYC184, P24E8-*hdeB*, Cm^R^ | This study |
| pAh2 | pACYC184, P10G6-*hdeB*, Cm^R^ | This study |
| pAh3 | pACYC184, P77C4-*hdeB*, Cm^R^ | This study |
| pAh4 | pACYC184, P50G1-*hdeB*, Cm^R^ | This study |
| pAs1 | pACYC184, P24E8-*sodB*, Cm^R^ | This study |
| pAs2 | pACYC184, P10G6-*sodB*, Cm^R^ | This study |
| pAs3 | pACYC184, P77C4-*sodB*, Cm^R^ | This study |
| pAs4 | pACYC184, P50G1-*sodB*, Cm^R^ | This study |
| pAk1 | pACYC184, P24E8-*katE*, Cm^R^ | This study |
| pAk2 | pACYC184, P10G6-*katE*, Cm^R^ | This study |
| pAk3 | pACYC184, P77C4-*katE*, Cm^R^ | This study |
| pAk4 | pACYC184, P50G1-*katE*, Cm^R^ | This study |
| pAks1 | pACYC184, P24E8-*katE*-*sodB*, Cm^R^ | This study |
| pAks3 | pACYC184, P77C4-*katE*-*sodB*, Cm^R^ | This study |
| pAks4 | pACYC184, P50G1-*katE*-*sodB*, Cm^R^ | This study |
| pAcc1 | pACYC184, f1-*ccdB*-f5, Cm^R^ | This study |
| pAcc2 | pACYC184, f2-*ccdB*-f5, Cm^R^ | This study |
| pMg1 | pMD19T, P24E8-*gadE*, Amp^R^ | This study |
| pMg2 | pMD19T, P10G6-*gadE*, Amp^R^ | This study |
| pMg3 | pMD19T, P77C4-*gadE*, Amp^R^ | This study |
| pMg4 | pMD19T, P50G1-*gadE*, Amp^R^ | This study |
| pMh1 | pMD19T, P24E8-*hdeB*, Amp^R^ | This study |
| pMh2 | pMD19T, P10G6-*hdeB*, Amp^R^ | This study |
| pMh3 | pMD19T, P77C4-*hdeB*, Amp^R^ | This study |
| pMh4 | pMD19T, P50G1-*hdeB*, Amp^R^ | This study |
| pMs1 | pMD19T, P24E8-*sodB*, Amp^R^ | This study |
| pMs2 | pMD19T, P10G6-*sodB*, Amp^R^ | This study |
| pMs3 | pMD19T, P77C4-*sodB*, Amp^R^ | This study |
| pMs4 | pMD19T, P50G1-*sodB*, Amp^R^ | This study |
| pMk1 | pMD19T, P24E8-*katE*, Amp^R^ | This study |
| pMk2 | pMD19T, P10G6-*katE*, Amp^R^ | This study |
| pMk3 | pMD19T, P77C4-*katE*, Amp^R^ | This study |
| pMk4 | pMD19T, P50G1-*katE*, Amp^R^ | This study |
| 1124 | pACYC184, P24E8-*gadE*-P24E8-*hdeB*-P10G6-*sodB*-P50G1-*katE*, Cm^R^ | This study |
| 414 | pACYC184, P50G1-*hdeB*-P24E8-*sodB*-P50G1-*katE*, Cm^R^ | This study |
| 2121 | pACYC184, P10G6-*gadE*-P24E8-*hdeB*-P10G6-*sodB*-P24E8-*katE*, Cm^R^ | This study |
| 3124 | pACYC184, P77C4-*gadE*-P24E8-*hdeB*-P10G6-*sodB*-P50G1-*katE*, Cm^R^ | This study |
| 1344 | pACYC184, P24E8-*gadE*-P77C4-*hdeB*-P50G1-*sodB*-P50G1-*katE*, Cm^R^ | This study |
| 3331 | pACYC184, P77C4-*gadE*-P77C4-*hdeB*-P77C4-*sodB*-P24E8-*katE*, Cm^R^ | This study |
| ^1^ Amp^R^, ampicillin resistance; Cm^R^, chloramphenicol resistance. | |  |

**Table S5** Primers used in this study.

| Primer names | Sequence (5’-3’) | Description |
| --- | --- | --- |
| Pasrori lib-RFPgi1-F | CTGCTGGCTACCCTGTGGAA | PCR fragment for Gibson assembly to construct *asr* promoter library |
| Pasrori lib-RFPgi1-R | ACACTTCCGTGAGTGGTTGGTTTCAGNNNNNNNNGATATGTACAAACGCTG |  |
| Pasrori lib-RFPgi2-F | GAAACCAACCACTCACGGAAGTCTGCCATTCCCAGGATATAGTTATTTCAACGGCCCC | PCR fragment for Gibson assembly to construct *asr* promoter library |
| Pasrori lib-RFPgi2-R | TTCCACAGGGTAGCCAGCAGCATC |  |
| Pasr-comgi1-F | GACCGGGTCGAATTTGCTTTCG | PCR fragment for Gibson assembly to construct plasmids pAg1, pAg2, pAg3, pAg4, pAh1, pAh2, pAh3, pAh4, pAs1, pAs2, pAs3, pAs4, pAk1, pAk2, pAk3 and pAk4 |
| Pasr-comgi1-R | TGTCATACCCTCAATTTGT |  |
| Pasr-comgi2-F | CTCGAGAGGCATCAAATAAAACG | PCR fragment for Gibson assembly to construct plasmids pAg1, pAg2, pAg3, pAg4, pAh1, pAh2, pAh3, pAh4, pAs1, pAs2, pAs3, pAs4, pAk1, pAk2, pAk3 and pAk4 |
| Pasr-comgi2-R | GAAATTCGAAAGCAAATTCGACC |  |
| gadEgi-F | CAAATTGAGGGTATGACAATGATTTTTCTCATGACGAAAG | PCR fragment *gadE* |
| gadEgi-R | CCTTTCGTTTTATTTGATGCCTCTCGAGCTAAAAATAAGATGTGATAC |  |
| hdeBgi-F | CAAATTGAGGGTATGACAATGAATATTTCATCTCTCCG | PCR fragment *hdeB* |
| hdeBgi-R | CCTTTCGTTTTATTTGATGCCTCTCGAGTTAATTCGGCAAGTCATTAG |  |
| sodBgi-F | CAAATTGAGGGTATGACAATGTCATTCGAATTACCTG | PCR fragment *sodB* |
| sodBgi-R | CCTTTCGTTTTATTTGATGCCTCTCGAGTTATGCAGCGAGATTTTTC |  |
| katEgi-F | CAAATTGAGGGTATGACAATGTCGCAACATAACGAAAAG | PCR fragment *katE* |
| katEgi-R | CCTTTCGTTTTATTTGATGCCTCTCGAGTCAGGCAGGAATTTTGTC |  |
| RBS-sodBgi-F | CGCAGGTGTGGTTATGCTACTAGAGACGGCCCCGCAGTGGGGTT | PCR fragment for Gibson assembly to construct plasmids pAks1, pAks3 and pAks4 |
| RBS-sodBgi-R | GCCTTTCGTTTTATTTGATGCCTCTC |  |
| katE-sodBgi1-F | CGCTAGCGGAGTGTATACTGGCTTACTATGTT | PCR fragment for Gibson assembly to construct plasmids pAks1, pAks3 and pAks4 |
| katE-sodBgi1-R | CTCTAGTAGCATAACCACACCTGCGTCAGGCAGGAATTTTGTCAA |  |
| katE-sodBgi2-F | CTCGAGAGGCATCAAATAAAACG | PCR fragment for Gibson assembly to construct plasmids pAks1, pAks3 and pAks4 |
| katE-sodBgi2-R | TAAGCCAGTATACACTCCGCTAGC |  |
| BsaI-f1-f2-F | GGTCTCAAATGATTCAGCGTTTGTACATAT | PCR fragment for TA cloning to construct plasmids pMg1, pMg2, pMg3 and pMg4 |
| BsaI-f1-f2-R | GGTCTCAGGACTAGATATGACGACAGGAAG |  |
| BsaI-f2-f3-F | GGTCTCAGTCCATTCAGCGTTTGTACATAT | PCR fragment for TA cloning to construct plasmids pMs1, pMs2, pMs3 and pMs4 |
| BsaI-f2-f3-R | GGTCTCAAGATTAGATATGACGACAGGAAG |  |
| BsaI-f3-f4-F | GGTCTCAATCTATTCAGCGTTTGTACATAT | PCR fragment for TA cloning to construct plasmids pMk1, pMk2, pMk3 and pMk4 |
| BsaI-f3-f4-R | GGTCTCAAAGCTAGATATGACGACAGGAAG |  |
| BsaI-f4-f5-F | GGTCTCAGCTTATTCAGCGTTTGTACATAT | PCR fragment for TA cloning to construct plasmids pMh1, pMh2, pMh3 and pMh4 |
| BsaI-f4-f5-R | GGTCTCAACTCTAGATATGACGACAGGAAG |  |

**Table S6** Sequence of DNA fragments or genes used in this study.

| Names | Sequence (5’-3’) | Description |
| --- | --- | --- |
| DNA fragments or genes | | |
| *asr* promoter (wild type) with native SD sequence | ATTCAGCGTTTGTACATATCGTTACACGCTGAAACCAACCACTCACGGAAGTCTGCCATTCCCAGGGATATAGTTATTTCAACGGCCCCGCAGTGGGGTTAAATGAAAAAACAAATTGAGGGTATGACA | 129 bp |
|  |  |  |
| *mCherry* | ATGGTTTCTAAAGGTGAAGAAGACAACATGGCTATCATCAAAGAATTTATGCGTTTCAAAGTTCACATGGAAGGTTCTGTGAACGGTCACGAATTTGAAATCGAAGGTGAAGGTGAAGGTCGTCCGTATGAAGGCACCCAGACCGCTAAACTGAAAGTTACCAAAGGTGGTCCGCTGCCGTTCGCTTGGGACATCCTGTCTCCGCAGTTCATGTACGGTTCTAAAGCGTATGTTAAACACCCGGCTGACATCCCGGACTACCTGAAACTGTCTTTCCCGGAAGGTTTCAAATGGGAACGTGTTATGAACTTTGAAGACGGTGGTGTTGTTACCGTTACCCAGGACTCTTCTCTGCAAGACGGTGAATTTATCTACAAAGTTAAACTGCGTGGCACCAACTTCCCGTCTGACGGTCCGGTTATGCAGAAGAAAACGATGGGTTGGGAAGCGTCTTCTGAACGTATGTACCCGGAAGACGGTGCTCTGAAAGGTGAAATCAAACAGCGTCTGAAACTGAAAGACGGTGGTCACTACGACGCTGAAGTTAAAACCACCTACAAAGCTAAAAAGCCGGTTCAACTGCCGGGTGCTTACAACGTGAACATCAAACTGGACATCACCTCTCACAACGAAGACTACACCATCGTTGAACAGTACGAACGTGCTGAAGGTCGTCACTCTACCGGCGGTATGGACGAACTGTATAAATAA | 711 bp |
|  |  |  |
| *ccdB* | ATGCAGTTTAAGGTTTACACCTATAAAAGAGAGAGCCGTTATCGTCTGTTTGTGGATGTACAGAGTGATATTATTGACACGCCCGGGCGACGGATGGTGATCCCCCTGGCCAGTGCACGTCTGCTGTCAGATAAAGTCTCCCGTGAACTTTACCCGGTGGTGCATATCGGGGATGAAAGCTGGCGCATGATGACCACCGATATGGCCAGTGTGCCGGTGTCCGTTATCGGGGAAGAAGTGGCTGATCTCAGCCACCGCGAAAATGACATCAAAAACGCCATTAACCTGATGTTCTGGGGAATATAA | 306 bp |
|  |  |  |
| *gadE* | ATGATTTTTCTCATGACGAAAGATTCTTTTCTTTTACAGGGCTTTTGGCAGTTGAAAGATAATCACGAAATGATAAAAATCAATTCCCTGTCAGAGATCAAAAAAGTAGGCAATAAACCCTTCAAGGTTATCATTGATACCTATCACAATCATATCCTTGATGAAGAAGCGATTAAATTTCTGGAGAAATTAGATGCCGAGAGAATTATTGTTTTGGCACCTTATCACATCAGTAAACTAAAAGCTAAAGCGCCTATTTATTTTGTTAGCCGCAAAGAAAGTATCAAAAATCTTCTTGAGATTACTTATGGTAAACACTTGCCCCATAAGAATTCACAATTATGTTTTTCACATAATCAGTTCAAAATTATGCAACTGATTCTGAAAAATAAAAATGAAAGCAATATCACGTCGACGCTCAATATTTCGCAACAAACATTAAAGATTCAGAAATTCAACATTATGTACAAGCTGAAACTAAGACGTATGAGCGACATCGTCACCCTGGGTATCACATCTTATTTTTAG | 528 bp |
|  |  |  |
| *hdeB* | ATGAATATTTCATCTCTCCGTAAAGCGTTTATTTTTATGGGCGCTGTAGCGGCTTTGTCACTGGTGAACGCACAATCTGCGTTGGCAGCCAATGAATCCGCTAAAGATATGACCTGCCAGGAATTTATTGATCTGAATCCAAAAGCAATGACCCCGGTTGCATGGTGGATGCTGCATGAAGAAACAGTATATAAAGGTGGCGATACCGTTACTTTAAATGAAACCGATCTCACTCAAATTCCTAAAGTGATCGAATACTGTAAGAAAAACCCGCAGAAAAATTTGTATACCTTCAAAAATCAAGCATCTAATGACTTGCCGAATTAA | 327 bp |
|  |  |  |
| *sodB* | ATGTCATTCGAATTACCTGCACTACCATATGCTAAAGATGCTCTGGCACCGCACATTTCTGCGGAAACCATCGAGTATCACTACGGCAAGCACCATCAGACTTATGTCACTAACCTGAACAACCTGATTAAAGGTACCGCGTTTGAAGGTAAATCACTGGAAGAGATTATTCGCAGCTCTGAAGGTGGCGTATTCAACAACGCAGCTCAGGTCTGGAACCATACTTTCTACTGGAACTGCCTGGCACCGAACGCCGGTGGCGAACCGACTGGAAAAGTCGCTGAAGCTATCGCCGCATCTTTTGGCAGCTTTGCCGATTTCAAAGCGCAGTTTACTGATGCAGCGATCAAAAACTTTGGTTCTGGCTGGACCTGGCTGGTGAAAAACAGCGATGGCAAACTGGCTATCGTTTCAACCTCTAACGCGGGTACTCCGCTGACCACCGATGCGACTCCGCTGCTGACCGTTGATGTCTGGGAACACGCTTATTACATCGACTATCGCAATGCACGTCCTGGCTATCTGGAGCACTTCTGGGCGCTGGTGAACTGGGAATTCGTAGCGAAAAATCTCGCTGCATAA | 582 bp |
|  |  |  |
| *katE* | ATGTCGCAACATAACGAAAAGAACCCACATCAGCACCAGTCACCACTACACGATTCCAGCGAAGCGAAACCGGGGATGGACTCACTGGCACCTGAGGACGGCTCTCATCGTCCAGCGGCTGAACCAACACCGCCAGGTGCACAACCTACCGCCCCAGGGAGCCTGAAAGCCCCTGATACGCGTAACGAAAAACTTAATTCTCTGGAAGACGTACGCAAAGGCAGTGAAAATTATGCGCTGACCACTAATCAGGGCGTGCGCATCGCCGACGATCAAAACTCACTGCGTGCCGGTAGCCGTGGTCCAACGCTGCTGGAAGATTTTATTCTGCGCGAGAAAATCACCCACTTTGACCATGAGCGCATTCCGGAACGTATTGTTCATGCACGCGGATCAGCCGCTCACGGTTATTTCCAGCCATATAAAAGCTTAAGCGATATTACCAAAGCGGATTTCCTCTCAGATCCGAACAAAATCACCCCAGTATTTGTACGTTTCTCTACCGTTCAGGGTGGTGCTGGCTCTGCTGATACCGTGCGTGATATCCGTGGCTTTGCCACCAAGTTCTATACCGAAGAGGGTATTTTTGACCTCGTTGGCAATAACACGCCAATCTTCTTTATCCAGGATGCGCATAAATTCCCCGATTTTGTTCATGCGGTAAAACCAGAACCGCACTGGGCAATTCCACAAGGGCAAAGTGCCCACGATACTTTCTGGGATTATGTTTCTCTGCAACCTGAAACTCTGCACAACGTGATGTGGGCGATGTCGGATCGCGGCATCCCCCGCAGTTACCGCACCATGGAAGGCTTCGGTATTCACACCTTCCGCCTGATTAATGCCGAAGGGAAGGCAACGTTTGTACGTTTCCACTGGAAACCACTGGCAGGTAAAGCCTCACTCGTTTGGGATGAAGCACAAAAACTCACCGGACGTGACCCGGACTTCCACCGCCGCGAGTTGTGGGAAGCCATTGAAGCAGGCGATTTTCCGGAATACGAACTGGGCTTCCAGTTGATTCCTGAAGAAGATGAATTCAAGTTCGACTTCGATCTTCTCGATCCAACCAAACTTATCCCGGAAGAACTGGTGCCCGTTCAGCGTGTCGGCAAAATGGTGCTCAATCGCAACCCGGATAACTTCTTTGCTGAAAACGAACAGGCGGCTTTCCATCCTGGGCATATCGTGCCGGGACTGGACTTCACCAACGATCCGCTGTTGCAGGGACGTTTGTTCTCCTATACCGATACACAAATCAGTCGTCTTGGTGGGCCGAATTTCCATGAGATTCCGATTAACCGTCCGACCTGCCCTTACCATAATTTCCAGCGTGACGGCATGCATCGCATGGGGATCGACACTAACCCGGCGAATTACGAACCGAACTCGATTAACGATAACTGGCCGCGCGAAACACCGCCGGGGCCGAAACGCGGCGGTTTTGAATCATACCAGGAGCGCGTGGAAGGCAATAAAGTTCGCGAGCGCAGCCCATCGTTTGGCGAATATTATTCCCATCCGCGTCTGTTCTGGCTAAGTCAGACGCCATTTGAGCAGCGCCATATTGTCGATGGTTTCAGTTTTGAGTTAAGCAAAGTCGTTCGTCCGTATATTCGTGAGCGCGTTGTTGACCAGCTGGCGCATATTGATCTCACTCTGGCCCAGGCGGTGGCGAAAAATCTCGGTATCGAACTGACTGACGACCAGCTGAATATCACCCCACCTCCGGACGTCAACGGTCTGAAAAAGGATCCATCCTTAAGTTTGTACGCCATTCCTGACGGTGATGTGAAAGGTCGCGTGGTAGCGATTTTACTTAATGATGAAGTGAGATCGGCAGACCTTCTGGCCATTCTCAAGGCGCTGAAGGCCAAAGGCGTTCATGCCAAACTGCTCTACTCCCGAATGGGTGAAGTGACTGCGGATGACGGTACGGTGTTGCCTATAGCCGCTACCTTTGCCGGTGCACCTTCGCTGACGGTCGATGCGGTCATTGTCCCTTGCGGCAATATCGCGGATATCGCTGACAACGGCGATGCCAACTACTACCTGATGGAAGCCTACAAACACCTTAAACCGATTGCGCTGGCGGGTGACGCGCGCAAGTTTAAAGCAACAATCAAGATCGCTGACCAGGGTGAAGAAGGGATTGTGGAAGCTGACAGCGCTGACGGTAGTTTTATGGATGAACTGCTAACGCTGATGGCAGCACACCGCGTGTGGTCACGCATTCCTAAGATTGACAAAATTCCTGCCTGA | 2262 bp |
|  |  |  |
| *rrnB* terminator | AGGCATCAAATAAAACGAAAGGCTCAGTCGGAAGACTGGGCCTTTCGTTTTATCTGTTGTTTGTCGGTGAACGCTCTCCTGAGTAGGACAAATCCGCCGGGAGCGGATTTGAACGTTGCGAAGCAACGGCCCGGAGGGTGGCGGGCAGGACGCCCGCCATAAACTGCCAGGCATCAAATTAAGCAGAAGGCCATCCTGACGGATGGCCTTTTTGCGTTTCTACAAACTCTTCCTGTCGTCATATCTA | 247 bp |

**Table S7** Sequences of the mutated region at *asr* promoter variants in this study with relative strength at pH 5.0 and pH response ratio.

| Names | Sequence of mutated region (5’-3’) | Relative strength to wild type *asr* promoter at pH 5.0 | pH response ratio |
| --- | --- | --- | --- |
| *asr* promoter variants used in acid-tolerance modules | | | |
| P24E8 | CACGTGAAG | 133% | 2.38 |
| P10G6 | GAGCCACGC | 100% | 1.83 |
| P77C4 | AGGGATTAA | 77% | 1.83 |
| P50G1 | ACTGAAAAA | 22% | 1.91 |
| Wild type *asr* promoter and other variants in *asr* promoter library | | | |
| Wild type *asr* promoter | TCCCAGGGA | 100% | 1.95 |
| P23C3 | TGTATTAAT | 126% | 3.21 |
| P21D7 | CAAGCCGTC | 123% | 1.95 |
| P76C8 | AAACTAAAT | 119% | 2.09 |
| P86A9 | CATTATACG | 108% | 1.82 |
| P86A3 | AGACGCATC | 108% | 1.81 |
| P91A10 | CAGCCGCTG | 106% | 1.86 |
| P72C7 | CTGAACGGA | 106% | 2.13 |
| P9E4 | CTTACACAC | 105% | 1.84 |
| P92C12 | TCTTGTCGG | 105% | 2.00 |
| P11G2 | GATCAAGAA | 105% | 1.87 |
| P20H4 | ATCAACAGA | 102% | 1.97 |
| P27A9 | ACAGCTCGA | 102% | 2.04 |
| P20H6 | TGTATTAAT | 102% | 2.12 |
| P5C12 | GCCAATGAT | 98% | 1.91 |
| P11G11 | AAGAAAAGG | 96% | 1.84 |
| P9E8 | GAAGCATAT | 95% | 1.80 |
| P20F12 | AACCAATAA | 93% | 1.85 |
| P9G8 | CACCCAAAC | 90% | 1.79 |
| P1F2 | CTTAGACAA | 88% | 2.03 |
| P11C8 | CCGTAAGAA | 87% | 1.82 |
| P71E8 | CCTAACAAA | 87% | 2.23 |
| P79E6 | GAAAAAAAA | 83% | 2.32 |
| P77C1 | TTAGAATTT | 82% | 1.98 |
| P15C1 | CAGCACTAA | 81% | 1.99 |
| P62E1 | ATAGACTCA | 79% | 1.90 |
| P17C3 | ACTGATAGA | 78% | 1.92 |
| P4D10 | TGTAATAAA | 78% | 2.02 |
| P82E1 | CGGGGTTCG | 75% | 1.86 |
| P85A7 | AGTGGAACC | 75% | 2.07 |
| P7G9 | TGAAAATTA | 70% | 1.91 |
| P10G9 | TATGACAAA | 67% | 1.91 |
| P16G11 | ATGTAAATT | 66% | 1.90 |
| P5C11 | TGCATATCA | 61% | 1.93 |
| P82C8 | ACTAACCTA | 61% | 1.83 |
| P88C3 | CGTTGAAAA | 60% | 1.92 |
| P6H2 | GCTAAAATT | 57% | 1.90 |
| P82A10 | ACTGAAAAT | 46% | 1.86 |
| P68G4 | CACAAAAAA | 38% | 2.19 |
| P96A11 | CGGGAAAAA | 36% | 2.06 |
| P82E8 | GGTGGTCGA | 34% | 1.79 |
| P25A12 | TATGAAAAA | 29% | 1.84 |
| P88G11 | TAGGAAAAA | 29% | 1.8 |
| P50A7 | ATTGAAAAA | 27% | 2.44 |
| P81E10 | ATCCAAAAA | 25% | 2.20 |
| P36E5 | ACTCAGAAA | 23% | 1.82 |
